# Atmospheric Effects on InSAR Measurements and Their Mitigation

**DOI:** 10.3390/s8095426

**Published:** 2008-09-03

**Authors:** Xiao-li Ding, Zhi-wei Li, Jian-jun Zhu, Guang-cai Feng, Jiang-ping Long

**Affiliations:** 1 Department of Land Surveying and Geo-Informatics, The Hong Kong Polytechnic University, Hung Hom, Kowloon, Hong Kong, China; E-mails: guangcai.feng@polyu.edu.hk; lsjplong@polyu.edu.hk; 2 School of Info-Physics and Geomatics Engineering, Central South University, Changsha 410083, Hunan, China; E-mails: zwli@mail.csu.edu.cn; zjj@mail.csu.edu.cn

**Keywords:** Interferometric Synthetic Aperture Radar (InSAR), atmospheric effects, atmospheric correction, MODIS, MERIS

## Abstract

Interferometric Synthetic Aperture Radar (InSAR) is a powerful technology for observing the Earth surface, especially for mapping the Earth's topography and deformations. InSAR measurements are however often significantly affected by the atmosphere as the radar signals propagate through the atmosphere whose state varies both in space and in time. Great efforts have been made in recent years to better understand the properties of the atmospheric effects and to develop methods for mitigating the effects. This paper provides a systematic review of the work carried out in this area. The basic principles of atmospheric effects on repeat-pass InSAR are first introduced. The studies on the properties of the atmospheric effects, including the magnitudes of the effects determined in the various parts of the world, the spectra of the atmospheric effects, the isotropic properties and the statistical distributions of the effects, are then discussed. The various methods developed for mitigating the atmospheric effects are then reviewed, including the methods that are based on PSInSAR processing, the methods that are based on interferogram modeling, and those that are based on external data such as GPS observations, ground meteorological data, and satellite data including those from the MODIS and MERIS. Two examples that use MODIS and MERIS data respectively to calibrate atmospheric effects on InSAR are also given.

## 1. Introduction

Synthetic Aperture Radar (SAR) Interferometry, commonly referred to as InSAR, IFSAR or SARI, is a synthesis of the SAR and the interferometry techniques [1]. InSAR is a powerful technology for topographic and ground surface deformation mapping due to its all-weather and day-and-night imaging capability, wide spatial coverage, fine resolution, and high measurement accuracy. Rogers and Ingalls [2] reported the first application of radar interferometry in Earth-based observations of Venus, while Graham [3] was regarded as the the first to apply an InSAR system to Earth topographic mapping. Airborne and spaceborne InSAR systems were then applied to Earth observation by Zebker and Goldstein [4] and Goldstein *et al.* [5], respectively. Gabriel *et al.* [6] first demonstrated the potential of differential InSAR (DInSAR) for centimeter or sub-centimeter level surface deformation mapping over a large area.

Significant progress has been made in further developing InSAR technology in the past two decades with the availability of a vast amount of globally covering SAR images from, e.g., ERS, Radarsat, JERS, Envisat, ALOS and TerraSAR sensors and with a wide range of applications of the technology (e.g., [7-19]). It is expected that InSAR will play a wider and more important role in both research and applications in the future with the advances of the technology and many ambitious SAR missions planed.

InSAR technology, however, has also limitations. One of the most intractable is the effect of the atmosphere (mainly the troposphere and the ionosphere) on repeat-pass InSAR. It is well known that electromagnetic waves are delayed (slowed down) when they travel through the troposphere. The effect often introduces significant errors to repeat-pass InSAR measurements. Massonnet *et al.* [8] first identified such effects. Since then, some intensive research has been carried out aiming to better understand and mitigate the effects. Zebker *et al.* [20] reported, for example, that spatial and temporal changes of 20% in the relative humidity of the troposphere could lead up to 10 to 14 cm errors in the measured ground deformations and 80 to 290 m errors in derived topographic maps for baselines ranging from 100 m to 400 m in the case of the SIR-C/X-SAR. A number of researchers have concluded that the tropospheric effects are a limiting factor for wide spread applications of repeat-pass InSAR (e.g., [11, 21-23]).

Contrary to the effects of the troposphere, the ionosphere tends to accelerate the phases of electromagnetic waves when they travel through the medium. The zenith ionospheric range error is proportional to the total electron content (TEC) in the ionosphere. For example, for C-band SAR, a TEC of 1 × 10^16^ m^-2^ causes a phase shift of about half a cycle [28]. The ionosphere is however a dispersive medium affecting the radar signals proportionately to the square of the wavelength [83]. For example, if the ionosphere causes 1.5 m range errors to the C-band (wavelength = 5.6 cm) signals, it will cause about 24 m range errors to the L-band (wavelength = 23 cm) signals if the same imaging geometry and atmospheric conditions are assumed. Since there are only very limited published works available on the ionospheric effects on InSAR, we will limit our discussions to the tropospheric effects hereafter. We will review systematically the work carried out in studying the atmospheric, especially the tropospheric effects on InSAR. The basic principles of the atmospheric effects on repeat-pass InSAR are first introduced. Research results on the properties of the atmospheric effects will then be examined. The various methods developed for mitigating the atmospheric effects will finally be studied.

## 2. Repeat-Pass SAR Interferometry

InSAR can be classified into across- and along-track interferometry according to the interferometric baseline formed, or single- and repeat-pass interferometry according to the number of platform passes involved. Two antennas are mounted on the same platform in along-track interferometry and a single platform pass suffices [24]. Across-track interferometry can be performed either with a one-antenna (e.g., ERS, Envisat) or a two-antenna (e.g., SRTM) SAR system. Revisit to the same scene is required for a one-antenna SAR system so that this is called repeat-pass SAR interferometry [25]. The atmospheric effects in the single-pass interferometry are basically removed completely in the interferometric computation as the effects are almost the same for the two SAR images. In repeat-pass interferometry, however, the atmospheric effects can become significant as the atmospheric conditions can vary considerably between the two SAR acquisitions. We will hereinafter limit our discussions to repeat-pass InSAR only.

**Figure 1.**
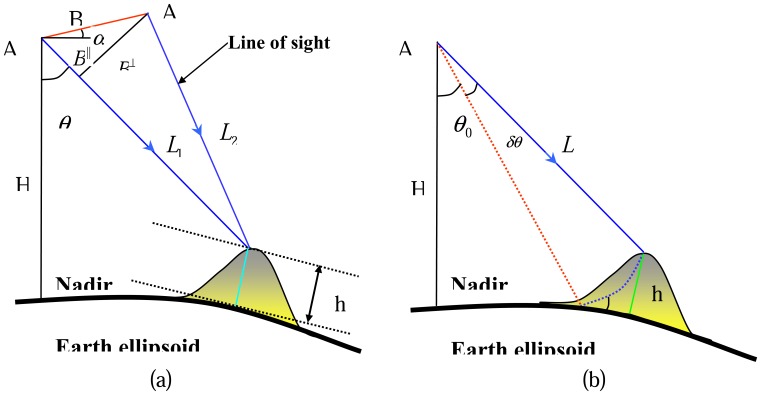
Interferometric geometry (from Li *et al.* [23]).

The geometrical configuration of repeat-pass SAR interferometry is illustrated in Figure 1a. A1 and A2 are the positions of radar platforms corresponding to the two acquisitions. The phases, *ψ*_1_ and *ψ*_2_, measured at the two platform positions to a ground point are:

(1) $$\begin{array}{cc} \psi_{1}=\frac{4\pi }{\lambda }L_{1}, & \psi_{2}=\frac{4\pi }{\lambda }L_{2} \end{array}$$

where *L*_1_ and *L*_2_ are the slant ranges and *λ* is the wavelength of the radar signal. The interferometric phase *ϕ* is then

(2) $$\phi =\psi_{1}-\psi_{2}=\frac{4\pi }{\lambda }\left(L_{1}-L_{2}\right)$$

Under the far field approximation, one gets

(3) $$\phi =\psi_{1}-\psi_{2}\approx \frac{4\pi }{\lambda }B^{∥}=\frac{4\pi }{\lambda }B\sin\left(\theta -\alpha \right)$$

where *α* is the orientation angle of the baseline and *θ* is the look angle.

When assuming a surface without topographic relief as illustrated in Figure 1b, the interferometric phase becomes [11]

(4) $$\phi_{0}=\frac{4\pi }{\lambda }B\sin\left(\theta_{0}-\alpha \right)$$

where *θ*_0_ is the look angle. If topographic relief is present, the look angle will differ from *θ*_0_ by *δθ*,

(5) $$\phi =\frac{4\pi }{\lambda }B\sin\left(\theta_{0}+\delta \theta -\alpha \right)$$

Combining Equations (4) and (5), we get the “flattened” phase

(6) $$\phi_{\text{flat}}=\phi -\phi_{0}\approx \frac{4\pi }{\lambda }B\cos\left(\theta_{0}-\alpha \right)\delta \theta =\frac{4\pi }{\lambda }B^{⊥}\delta \theta$$

The relationship between the topographic height and *δθ* can be easily established (see Figure 1b)

(7) $$h\approx L\delta \theta_{0}\cdot \sin\theta_{0}$$

Thus the topographic height can be expressed as

(8) $$h=\frac{\lambda L}{4\pi B}\frac{\sin\theta_{0}}{\cos\left(\theta_{0}-\alpha \right)}\phi_{\text{flat}}$$

**Figure 2.**
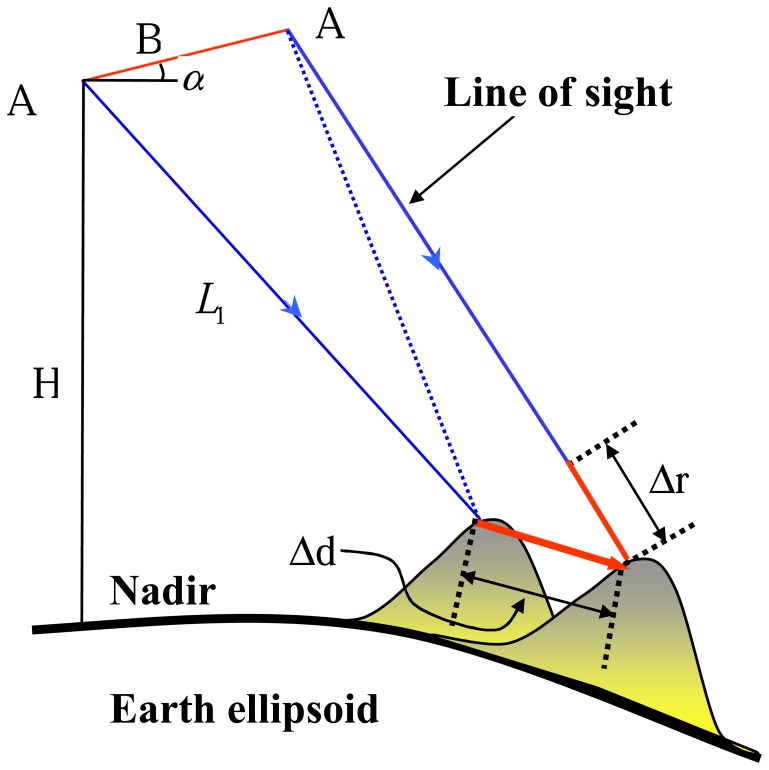
Geomtry of DInSAR.

The aforementioned process of topography reconstruction is based on the assumption that the imaged surface is stationary during the acquisitions. The interferometric phase in repeat-pass interferometry in fact measures any ground displacement in addition to topography. DInSAR is the technique to extract displacement signature from a SAR interferogram over the acquisition period. In Figure 2, there is an exaggerated ground displacement Δd between the two acquisitions whose projection onto radar line-of-sight (LOS) direction is Δr.

The displacement will introduce a variation of interferometric phase which is proportional to Δr:

(9) $$\Delta \phi_{\text{flat}}=\frac{4\pi }{\lambda }\Delta r$$

Therefore, the interferometric phase includes topography information as well as deformation information,

(10) $$\phi_{\text{flat}}\approx \frac{4\pi }{\lambda }\frac{B\cos\left(\theta_{0}-\alpha \right)}{L\sin\theta_{0}}h+\frac{4\pi }{\lambda }\Delta r$$

To map the ground deformation between two SAR acquistions, the topographic contribution must be removed. According to the ways to remove the topographic contribution, three types of DInSAR configuration can be distinguished: (1) two-pass plus external DEM, (2) three-pass, and (3) four-pass. In two-pass plus external DEM formulation, a SAR interferogram (topographic interferogram thereinafter) is simulated based on the DEM and the imaging geometry of the “real” interferogram (deformation interferogram thereinafter) and is removed from the deformation interferogram. However, in three-pass and four-pass formulations, both the topographic and the deformation interferograms are generated from SAR images. The only difference between them is that in three-pass interferometry, one image is shared by both the topographic and the deformation interferograms. The two-pass plus external DEM and three-pass and four-pass configuration DInSAR can be expressed as:

(11) $$\Delta r_{\text{two}}=\frac{\lambda }{4\pi }\left(\phi_{d}-\phi_{\text{sim},t}\right)$$

(12) $$\Delta r_{\text{three},\text{four}}=\frac{\lambda }{4\pi }(\phi_{d}-\frac{B_{d}^{⊥}}{B_{t}^{⊥}}\phi_{t})$$

where *ϕ_d_* and *ϕ_t_* are phases of deformation and topography interferograms, respectively, and $B_{d}^{⊥}$ and $B_{t}^{⊥}$ are perpendicular baseline components of the deformation and topography interferograms, respectively.

The interferometric phase in Equation (10) may also include linear phase ramps caused by orbital errors that should be modeled and removed to derive the ground deformation [22, 28]. This can at times become a problem when the deformation or topography phases also have linear trends. We will however not discuss this problem further in this paper.

## 3. The Atmosphere and its Effects on Repeat-Pass InSAR

Atmospheric artifacts in SAR interferograms are mainly due to changes in the refractive index of the medium. These changes are mainly caused by the atmospheric pressure, temperature and water vapor. In most cases, the spatial variations of pressure and temperature are not large enough to cause strong, localized phase gradients in SAR interferograms. Their effects are generally smaller in magnitude and more evenly distributed throughout the interferogram when comparing with that of the water vapor, and sometimes difficult to be distinguished from errors caused by orbit uncertainties [22, 26]. The artifact caused by localized water vapor generally dominates the atmosphere induced artifacts in SAR interferograms. Water vapor is mainly contained in the near-ground surface troposphere (up to about 2 km above ground), where a strong turbulent mixing process occurs. Turbulent mixing can result in three-dimensional (3D) spatial heterogeneity in the refractivity and can cause localized phase gradient in both flat and mountainous regions [27, 28]. Besides turbulent mixing, another atmospheric process with clear physical origin is the stratification of the atmosphere. Stratification of the atmosphere into layers of different vertical refractivity causes additional atmospheric delays in mountainous regions [27, 28]. It should be noted that although water vapor is often considered the most important parameter causing the tropospheric delays, there are cases, e.g., in regions with strong topography, changes in pressure between two acquisitions can generate a bigger tropospheric delay signal than humidity variation.

Clouds are formed when the water vapor in the air condenses into visible mass of droplets or frozen crystals. Clouds are divided into two general categories, layered and convective. These are named stratus clouds and cumulus clouds respectively. The liquid water content in the stratiform clouds is usually low so that they do not cause significant range errors to SAR signals. The liquid water content in the cumulus clouds can however range from 0.5 to 2.0 g/m^3^ and cause zenith delays of 0.7 to 3.0 mm/km [26], significant to InSAR measurements.

Due to the propagation delay of radar signals, in repeat-pass SAR interferometry systems, the phase measurements corresponding to Equation (1) becomes:

(13) $$\begin{array}{cc} \psi_{1}=\frac{4\pi }{\lambda }\left(L_{1}+\Delta L_{1}\right), & \psi_{2}=\frac{4\pi }{\lambda }\left(L_{2}+\Delta L_{2}\right) \end{array}$$

where Δ*L*_1_ and Δ*L*_2_ are atmospheric propagation delays of radar signals corresponding to the first and the second acquisitions. This gives the interferometric phase

(14) $$\phi =\psi_{1}-\psi_{2}=\frac{4\pi }{\lambda }\left(L_{1}-L_{2}\right)+\frac{4\pi }{\lambda }\left(\Delta L_{1}-\Delta L_{2}\right)$$

where $\frac{4\pi }{\lambda }\left(L_{1}-L_{2}\right)$ are topography and surface deformation induced interferometric phase, and $\frac{4\pi }{\lambda }\left(\Delta L_{1}-\Delta L_{2}\right)$ is the atmosphere induced interferometric phase. From Equation (14), we can see that the atmosphere induced phase errors are easily interpreted as topography or surface deformation.

It is obvious from Equation (14) that it is the relative tropospheric delay (Δ*L*_1_ − Δ*L*_2_) that causes errors in InSAR measurements. If the atmospheric profiles remain the same at the two acquisitions, the relative tropospheric delay will disappear. In addition, if Δ*L*_1_ − Δ*L*_2_ = *constant* for all the resolution cells in an area of interest, the atmospheric effects will also be cancelled out. The two conditions are, however, next to impossible to occur in practice. First, the troposphere, especially the tropospheric water vapor, varies significantly over periods of a few hours or shorter. It is, therefore, highly unlikely to have the same atmospheric profiles even over currently the shortest revisit interval of one day (for ERS-1/ERS-2). Second, it is also rather rare for the relative tropospheric delays to be constant for all the resolution cells due to local tropospheric turbulences, which affect flat terrain as well as mountainous terrain and to vertical stratification which only affects mountainous terrain [27-29].

The influences of the atmosphere induced phase errors on repeat-pass topographic and two-pass surface deformation measurements are straightforward [4, 9]

(15) $$\sigma_{h}=\frac{\lambda }{4\pi B^{⊥}}L\sin\theta \sigma_{\phi }$$

(16) $$\sigma_{\Delta r,\text{two}}=\frac{\lambda }{4\pi }\sigma_{\phi }$$

where *σ_ϕ_* is the phase error in the interferogram; *σ_h_* is the resultant height error; and *σ*_Δ_*_r,two_* is the deformation error for two-pass D-InSAR.

Assuming the same standard deviation *σ_ϕ_* on each interferogram, the covariance matrixes of *ϕ* = [*ϕ_d_ ϕ_t_*] *^T^* for three-pass and four-pass interferometry are:

(17) $$Cov_{\phi ,\text{three}}=\left[\begin{array}{cc} \sigma_{\phi }^{2} & \frac{1}{2}\sigma_{\phi }^{2}\\ \frac{1}{2}\sigma_{\phi }^{2} & \sigma_{\phi }^{2} \end{array}\right]$$

(18) $$Cov_{\phi ,\text{four}}=\left[\begin{array}{cc} \sigma_{\phi }^{2} & 0\\ 0 & \sigma_{\phi }^{2} \end{array}\right]$$

According to error propagation theorem, the effects of atmosphere induced phase errors on deformation mapping in three-pass and four-pass interferometry are:

(19) $$\sigma_{\Delta r,\text{three}}=\frac{\lambda }{4\pi }\sqrt{1-\frac{B_{d}^{⊥}}{B_{t}^{⊥}}+{\left(\frac{B_{d}^{⊥}}{B_{t}^{⊥}}\right)}^{2}}\sigma_{\phi }$$

(20) $$\sigma_{\Delta r,\text{four}}=\frac{\lambda }{4\pi }\sqrt{1+\left(\frac{B_{d}^{⊥}}{B_{t}^{⊥}}\right)}\sigma_{\phi }$$

## 4. Properties of Atmospheric Signals in SAR Interferograms

### 4.1 Atmospheric Signals from SAR Interferograms

A SAR interferogram is a superposition of information on the topography, the surface deformation between the two SAR acquisitions, the differential atmospheric propagation delays between the two SAR acquisitions, and various noise (e.g., [22, 26]). The contribution from the topography can be removed by using a reference elevation model. That from the surface deformation can be neglected or removed if the surface deformation of the study area between the two SAR acquisitions is insignificant or the deformation is known. In addition, multi-looking operations and careful interferometric processing can help to suppress the noise. Therefore at the end an interferogram that contains only the atmospheric signature can be obtained [26]. The atmospheric signature thus obtained is very useful for studying the properties of atmospheric effects on InSAR. Besides, the atmospheric signals can be used to derive various atmospheric products. For example, Hanssen et al. [30] used atmospheric signals derived from SAR interferograms to map high-resolution water vapor.

### 4.2 Anisotropic Properties of Atmospheric Signals

Radon transform is the projection of image intensities along a radial line at a specified angle. A single Radon transform is a mapping of an image from two dimensions to one dimension where the image intensities collapse to a profile. Radon transform is therefore a tool to investigate anisotropy in images since systematic intensity variations in a particular direction will be visible as a profile [31].

**Figure 3.**
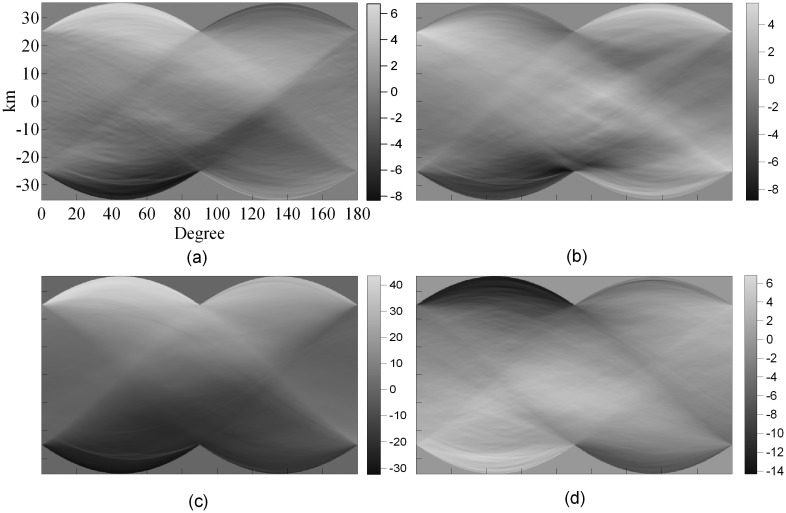
Radon transform of atmospheric signals in SAR pairs acquired on: (a) 19 and 20 February, 1996, (b) 25 and 26 March, 1996, (c) 3 and 4 June, 1996, and (d) 16 November and 21 December, 1999 (from Li *et al.* [33]).

Hanssen [26] first used Radon transform to examine the anisotropy of atmospheric signatures in SAR interferograms, while Jónsson [32] used it to characterize the anisotropy of the noise in SAR interferograms. Li *et al.* [33] used Radon transform to study the anisotropy of atmospheric signatures in four SAR interferograms over Shanghai. The results are shown in Figure 3.

The Radon transform of atmospheric signals showed varying degrees of anisotropy. For example, the first transform (Figure 3a) showed strong asymmetry especially for profiles of 0° to 90°. This implies that there are areas of very different atmospheric signals in the southwest and northeast corners of the interferogram. However, as the authors pointed out, none of the transforms showed complex variations in the signals, perhaps due to the fact that the studied region is very flat. The results are quite different from those obtained in mountainous regions where the atmospheric effects vary significantly (e.g., [34]) perhaps due to the vertical stratification or the “static” effect of the troposphere in the mountainous regions [29, 35, 43] and the effects of mountains on local weather conditions.

### 4.3 Gaussianity of Atmospheric Signals

It is important to examine the Gaussianity of atmospheric signals in SAR interferograms as different processing strategies must be applied for Gaussian and non-Gaussian signals. There are a number of hypothesis tests to study whether a signal is Gaussian or non-Gaussian. The Jarque-Bera test is based on classical measures of skewness and kurtosis and it examines whether the sample skewness and kurtosis are unusually different from their expected values [36]. The Hinich test is however a frequency domain test that examines the deviation of the bispectrum of the signal from zero as the bispectrum of a Gaussian signal is zero [37]. Li et al. [33] used both the Jarque-Bera and the Hinich methods to test the atmospheric signals in the four SAR interferograms over Shanghai. The results from both of the methods indicate that the atmospheric signals in all the interferograms are non-Gaussian.

### 4.4 Spectral Characteristics of Atmospheric Signals

The spectrum of atmospheric signals in a SAR interferogram reveals the energy distribution of the atmospheric effects at different spatial scales. Two-dimensional (2D) FFT is generally used to estimate the 2D power spectra of atmospheric signals. As the power spectra derived can be very noisy, the one-dimensional (1D) rotationally averaged power spectra are usually calculated from the 2D power spectra and used to study the energy distribution of atmospheric signals (e.g., [28, 38]).

**Figure 4.**
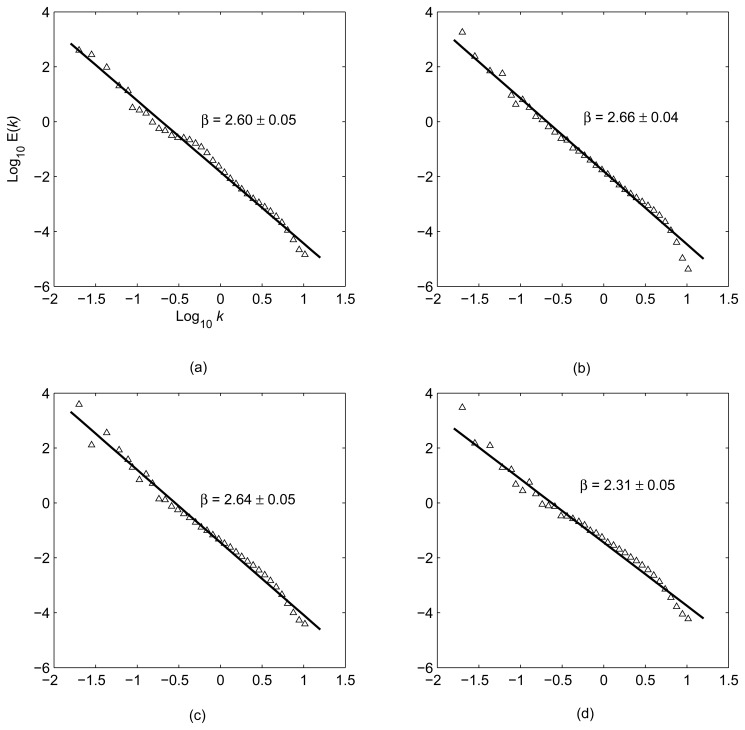
Power spectra of atmospheric signals for SAR pairs acquired on: (a) 19 and 20 February, 1996, (b) 25 and 26 March, 1996, (c) 3 and 4 June, 1996, and (d) 16 November and 21 December, 1999. (from Li *et al.* [33]).

Goldstein *et al.* [39] first calculated the power spectra of atmospheric signals in a SAR interferogram, and demonstrated that the spectra followed a power law distribution with a power exponent of −8/3. This feature is associated with Kolmogorov turbulences, indicating the nature of scale invariance or scaling [40]. Hanssen [26] analyzed the spectra of atmospheric signals in 26 SAR interferograms over Netherland. The results also showed the power law feature. Li *et al.* [33] calculated the power spectra of atmospheric signals for the four interferograms over Shanghai (Figure 4). It is very clear that the signals follow on the whole the power law distribution. The results are in good agreement with those presented for Mojave desert of California by Goldstein *et al.* [39] and for the Groningen and Flevoland area of Netherlands by Hanssen [28].

The power law spectral characteristics of the atmospheric signals are very useful. For example, Ferretti *et al.* [41] used the spectral characteristics to estimate the powers of thermal noise and atmospheric effects, and developed a method based on the results to combine SAR DEMs in wavelet domain. Ferretti et al. [42] also utilized the spectral characteristics to design filters to separate atmospheric effects from nonlinear subsidence. Williams *et al.* [43] considered that the low-frequency (long wavelength) components of atmospheric effects had larger energy so that sparse external data such as GPS and ground meteorological data can be used to calibrate the effects. Li et al. [44, 45] used the power law nature of the atmospheric effects in designing algorithms to model and correct the effects based on GPS and meteorological data.

The power law can be described by

(21) $$E(k)\propto k^{-\beta }$$

where *E*(*k*) is the power; *k* is the spatial frequency; and *β* is the power exponent. The power exponent is an important indicator of data stationarity. Theoretically, when 1 < *β* < 3, the data series are considered non-stationary but with stationary increments [28, 46]. The estimated spectral exponents range from 2.31 to 2.66 so that the signals have this property. Stationary increments lead to stationary structural function but do not imply that the variance and covariance of the atmospheric signals can be uniquely determined. Therefore, care should be taken when using InSAR data to constrain geophysical models, where the covariances of the noise are generally needed (e.g., [32, 38, 47]).

The 3D Kolmogorov tropospheric turbulence can occur within the region of up to several kilometers in elevation, usually referred to as the effective height of the wet troposphere. The LOS ranges of space-borne SAR systems are much larger than the effective height. The wet tropospheric delays can be therefore typically modeled as 2D turbulence [28].

The power exponent is an important parameter for estimating to what extent the atmospheric effects can be determined and removed with the help of external data. The accumulated energy of the atmospheric signals can be estimated based on the information for different scales by integrating the atmospheric power over the spatial frequencies. The spatial scales corresponding to 90% of the energy thus computed for the four interferograms mentioned above are 0.82, 1.01, 0.94, and 0.29 km, respectively [33]. The spatial scales can be considered as the lowest spatial resolution of external atmospheric data required to calibrate 90% of the atmospheric effects in the SAR interferograms. Therefore, to calibrate 90% of the atmospheric effects for the four interferograms, the spatial resolution of the external atmospheric data (assuming no measurement errors) must reach 0.82, 1.01, 0.94, and 0.29 km, respectively [33]. This can be a reference when one applies corrections for atmospheric effects on InSAR based on external data.

## 5. Mitigation of Atmospheric Effects on Repeat-Pass InSAR

### 5.1 Correction of Atmospheric Effects based on External data

#### 5.1.1 Correction of atmospheric effects based on ground meteorological observations

Using ground meteorological data to calibrate tropospheric delays in radio ranging has been well documented [48-52]. Hanssen and Feijt [53] and Zebker *et al.* [20] proposed the use of the Saastamoinen model to assess the potential effects of the troposphere on InSAR measurements. Delacourt *et al.* [29] presented a case study of correcting atmospheric errors by using meteorological observations at a reference point together with tropospheric delay models, vertical gradient models of the meteorological parameters, and the DEM of the study area. The results showed that tropospheric corrections reached 2 fringes for some interferograms, and that on average the accuracy of the interferograms was about ±1 fringe after the corrections were applied. Bonforte *et al.* [54] demonstrated congruence between the tropospheric zenith delays estimated from GPS observations and from tropospheric models and meteorological data. The results confirmed that the meteorological data could be applied to calibrate InSAR measurements like what had been done to correct GPS observations, and suggested a possible integration of the two data sources for improving models of the atmospheric effects. Li *et al.* [45] studied InSAR atmospheric correction by using meteorological observations, GPS observations, and both types of observations. The results showed that the integration of the observations produced better results.

The difficulties in using meteorological data to correct atmospheric effects on InSAR include mainly the poor accuracy of the atmospheric delays estimated from empirical tropospheric models and the usually very sparse distribution of meteorological stations.

#### 5.1.2 Correction of atmospheric effects based on GPS observations

The advances in GPS meteorology have enabled accurate estimation of tropospheric delays from GPS observations [55, 56], and have provided an opportunity to use GPS observations to evaluate and calibrate the atmospheric effects on InSAR measurements. However, the spatial resolution of GPS stations is in general much lower than that of InSAR data. This poses a potential limitation in applying GPS observations to correcting InSAR measurements.

Considering the power law nature of the atmospheric noise, Williams *et al.* [43] however dismissed the belief that the spatially sparse GPS observations (compared to the scales of the atmospheric irregularities and the resolutions of SAR data) were unsuitable for calibrating the atmospheric effects. Using simulated data, the authors demonstrated that in general it is possible to use sparsely distributed data to reduce the noise in a more densely distributed data set, and that in particular it is possible to use zenith delays estimated from GPS observations to reduce the atmospheric noise in InSAR measurements. Bock and Williams [57] reported through a cross validation analysis that using zenith delays estimated from GPS observations and the Kriging interpolator, more than 90% of the atmospheric delays at the unsampled points in a SAR image can be retrieved and therefore removed for the Los Angeles basin where fairly dense GPS stations had been in operation. On the other hand, only 40% of the atmospheric delays can be retrieved for regions outside the basin where the density of GPS stations is much lower. Also using cross validation analysis, Janssen *et al.* [58] tested the effectiveness of three interpolators, i.e., inverse distance weighting, Kriging and spline, in interpolating the GPS-derived atmospheric delays to the SAR resolution level and correcting the atmospheric effects on InSAR on a pixel-by-pixel basis. The results showed that the inverse distance weighting and Kriging interpolators are better than the spline interpolator. Webley *et al.* [59] proposed a procedure to use the water vapor delays derived from both the GPS observations and the non-hydrostatic three-dimensional (NH3D) meteorological model to calibrate the atmospheric effects on InSAR.

The research results in [43, 57-59] are mainly from cross validation analysis, but not from corrections to real SAR interferograms. Li *et al.* [44] recently proposed a new method and applied it to correct a SAR interferogram. In this method, an atmospheric delay map for each SAR acquisition is generated in two steps. First, a “mean” atmospheric delay map is calculated using the method adopted by Delacourt *et al.* [29]. Second, the “mean” atmospheric delay map is amended with the atmospheric zenith delays derived from a dense GPS network, mainly to calibrate the estimated “mean” atmospheric delays and to compensate their horizontal heterogeneity. Using 14 GPS stations over Mt. Etna, the authors corrected a SAR interferogram and achieved 27.2% overall improvement in the accuracy of the InSAR measurements. Based on the variance model of water vapor delays derived by Emardson *et al.* [47], a linear interpolator and the best linear unbiased estimator, Li *et al.* [60] developed a GPS topography-dependent turbulence model for InSAR atmospheric correction. Test results show that the model is much better than the inverse distance weighting interpolator.

There are many GPS networks around the world operated in continuous mode. If GPS observations prior to and after SAR acquisitions are available, they can also contribute to the correction of atmospheric effects on InSAR. Onn [62] and Onn and Zebker [61] applied this method to InSAR atmospheric correction based on Taylor's “frozen-flow” hypothesis. The results showed that additional improvement can be achieved when GPS observations prior to and after SAR acquisitions are added to the GPS-based InSAR atmospheric correction models.

The various methods proposed to date that use GPS observations to correct InSAR atmospheric effects differ primarily in the algorithms used to generate atmospheric delay maps from the spatially sparse GPS atmospheric delay measurements. Therefore, the accuracy of the corrections depends on how much atmospheric delays can be retrieved at the unsampled locations from the sparse GPS measurements. With the gradual increase in the density of GPS networks around the world, the method should become more and more useful.

#### 5.1.3 Correction of atmospheric effects based on high-resolution meteorological models

Numerical meteorological modeling is an essential tool in atmospheric research. Numerical meteorological modeling can be carried out on global, regional or mesoscale. The global and regional numerical meteorological models are usually too coarse to model atmospheric effects on InSAR. The mesoscale numerical models can have a continuous time scale and a horizontal spatial scale of a few kilometers, and are therefore suitable for InSAR atmospheric correction. Integrated water vapor content along the radar paths can be retrieved from such models and used to calibrate atmospheric effects on InSAR.

Wadge *et al.* [59] used the local-scale non-hydrostatic three-dimensional models (NH3D) to simulate the path delays due to water vapor over Mount Etna, and found that the NH3D delays were in general agree well with the ERS-2 SAR interferogram and the GPS estimates. Webley [65] and Webley *et al.* [64] tested correcting atmospheric effects on a descending and two ascending SAR interferograms over Mount Etna by using the path delays derived from the NH3D models. The results showed that the correction can result in up to 28.6% improvements in terms of the phase standard deviations. The accuracy improvement is however highly dependent on the data used to initialize the NH3D models. Foster *et al.* [66] used the MM5 models (a non-hydrostatic mesoscale meteorological model produced by the National Center for Atmospheric Research (NCAR)/Pennsylvania State University) to predict the atmospheric delay maps and then to correct 44 SAR interferograms over Hawaii. The results showed that on average atmospheric effects with wavelengths of 30 km or greater can be significantly reduced, while those with wavelengths shorter than 30 km cannot be effectively reduced. More recently, Puysségur *et al.* [67] found that water vapor content estimated from Envisat Medium Resolution Imaging Spectrometer (MERIS) and from MM5 model were consistent and unbiased, and thus proposed to integrate MM5 model and MERIS data for InSAR atmospheric correction. Test results showed that about 43% of the atmospheric signals can be removed. High-resolution meteorological models have offered some promising opportunities for mitigating atmospheric effects on InSAR although further research needs to be carried out to enhance the accuracy and reliability of the method.

#### 5.1.4 Correction of atmospheric effects based on MODIS data

The near-IR water vapor products provided by the Moderate Resolution Imaging Spectroradiometer (MODIS) have a spatial resolution of 1 km× 1 km (at nadir) and an accuracy of 5-10% [68]. The high-resolution water vapor products appear to be very useful for modeling and correcting atmospheric effects on InSAR although as an optical sensor MODIS measurements are sensitive to the presence of clouds. The resolutions of even the densest GPS networks in the world, e.g., the Southern California Integrated GPS Network (SCIGN), are more than ten times sparser than the resolution of MODIS.

Li *et al.* [69] first presented some results of using MODIS data to correct atmospheric effects on InSAR over Mount Etna and Los Angeles. Li *et al.* [70] proposed an integration of MODIS and GPS data for InSAR atmospheric correction, where the GPS data (more exactly the GPS precipitable water vapor (PWV) data) are mainly used to calibrate the MODIS PWV data. Experiments over Los Angeles area showed that the atmospheric singals in the SAR interferograms were significantly reduced with this method and the geophysical signals in the InSAR measurements became more prominent after the corrections were made.

Considering that all data interpolators unavoidably suffer from smoothing effects [71], Li [72] proposed a hybrid algorithm that jointly uses the Kriging interpolator and the conditional spectral simulation method to interpolate the MODIS PWV in correcting the atmospheric signals in SAR interferograms.

**Figure 5.**
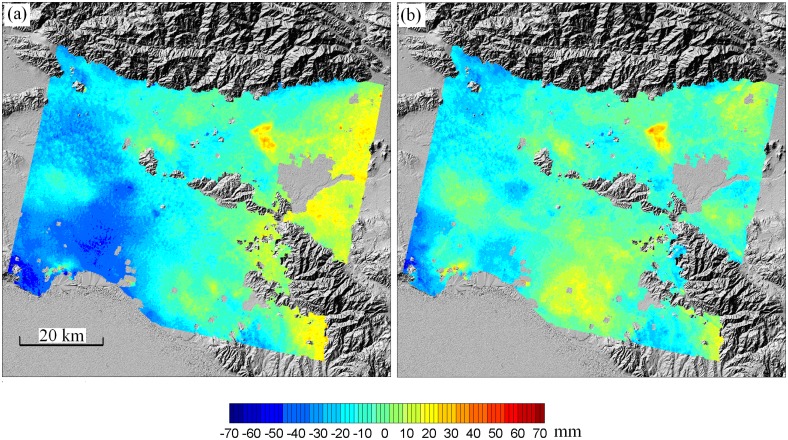
Atmospheric path delay corrections for interferometric pair of 29 July 2000 and 18 August 2001 over Los Angeles basin, South California. (a) original interferogram (deformation field has been modeled with GPS observations and removed from the interferogram); (b) interferogram corrected using MODIS data. (from Li [72]).

Figure 5 shows the original and the corrected ERS-2 interferogram over Los Angeles by using the MODIS data and the developed hybrid algorithm. Note that the original interferogram has been corrected for topographic phases with a known DEM and for deformation phases with GPS positioning results from SCIGN [72]. Thus, the signals left in the original interferogram can be considered solely from the atmospheric effects. After the corrections were applied, the negative atmospheric phases in the southwestern part of the interferogram and the positive atmospheric phases along the eastern margin of the interferogram were largely removed. The phases at the lower central part of the interferogram became however more significant, coupled with some under-modeled positive/negative residual phases in the corners of the interferogram. The phase standard deviation in the original deformation-free interferogram (Figure 5a) is 14.9 mm, while this becomes 10.6 mm in the corrected interferogram (Figure 5b), representing an improvement of 28.9% in the measurement accuracy.

The MODIS PWV measurements however are sensitive to the presence of clouds as noted earlier, which limits significantly the use of MODIS PWV in cloudy regions. In addition, systematic biases in space borne MODIS PWV measurements may exist and need to be calibrated with more accurate PWV measurements (e.g., GPS PWV).

#### 5.1.5 Correction of atmospheric effects based on MERIS data

The MERIS onboard the Envisat satellite allows for global retrieval of PWV every three days, with two near infrared water vapor channels. It therefore can acquire water vapor data simultaneously with the Advanced SAR (ASAR). Its PWV measurements have a resolution as high as 300 m and accuracy higher than that of MODIS [69]. MERIS measurements therefore offer an opportunity for the atmospheric effects on ASAR measurements to be accurately modeled.

Li *et al.* [73] assessed the potential of using MERIS near-infrared water vapor products to correct ASAR interferometric measurements. The MERIS and the GPS/radiosonde water vapor products tested agreed to each other to within 1.1 mm (standard deviation) on average. It was also pointed out that the major limitation with the use of MERIS water vapor products is the low frequencies of cloud free conditions, i.e., about 25% globally although for certain areas like Easter Tibet and Southern California, the frequencies can be much higher.

Using the Los Angeles area as an example, Li *et al.* [74] showed that MERIS water vapor data could significantly reduce atmospheric effects in SAR interferograms. After corrections were made with the MERIS data, the RMS difference between GPS and InSAR range changes in the satellite LOS direction decreased from 0.89 cm to 0.54 cm in one interferogram, and from 0.83 cm to 0.59 cm in another. Puysségur *et al.* [67] proposed the integration of MM5 simulated water vapor data and MERIS data for InSAR atmospheric correction, as noted earlier. However, no significant improvements were found by adding the MERIS data to the MM5 model.

**Figure 6.**
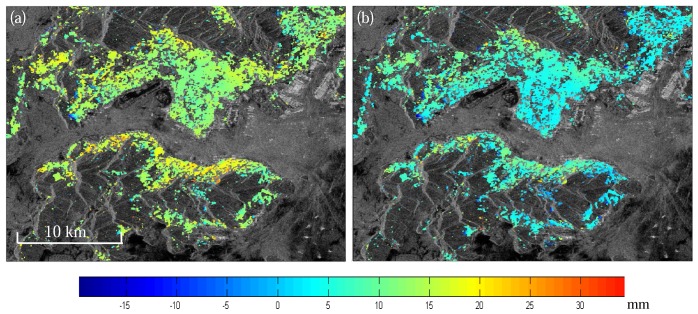
Atmospheric path delay correction for interferometric pair of 30 April 2006 and 11 March 2007 over Hong Kong area. (a) original interferogram; (b) interferogram corrected using MERIS data.

Figure 6 shows an example of correcting atmospheric effects on InSAR using MERIS data over Hong Kong region. The SAR images are acquired on 30 April 2006 and 11 March 2007, respectively. Topographic phases have been removed with a reference DEM. GPS positioning results have shown that there were no significant deformations during this period in region. The signals in Figure 6a can therefore be considered from atmospheric heterogeneity only. Figure 6b shows the corrected interferogram with reduced resolution (RR) MERIS water vapor data. It can be seen that the positive atmospheric phases in some of the areas have been significantly removed. The standard deviation of the phases in the original interferogram (Figure 6a) is 5.72 mm and that in the corrected interferogram (Figure 6b) is 4.51 mm, indicating an improvement of about 21% after the corrections were made.

### 5.2 Correction of Atmospheric Effects based on Correlation Analysis

There are mainly two types of correlation analysis adopted to reduce atmospheric effects on InSAR. The first type analyzes the correlation between interferograms, and the second the correlation between atmosphere-induced interferometric phases and elevation. Sarti *et al.* [75] proposed to characterize the atmospheric artifacts in SAR interferograms and to remove them through analyzing the correlation between interferograms. Fruneau and Sarti [76] proposed to separate the deformation signals from atmospheric artifacts by exploiting the correlation between interferograms. The method does not aim to remove atmospheric noise from a SAR interferogram, but manages to extract the common (correlated) deformation signals within two SAR interferograms through analyzing the correlation of the signals. Using this method, the authors successfully extracted the deformation signals from interferograms over Paris. Sarti *et al.* [77] compared this method with other methods for atmospheric effect mitigation like the stacking and the persistent scatterer (or permanent scatterer) InSAR (PSInSAR) method and pointed out the advantages of the correlation analysis method when the number of available SAR images is not large.

Beauducel *et al.* [35] proposed to separate deformation signals from atmospheric artifacts over Mount Etna by analyzing the correlation between the atmosphere-induced interferometric phases and the elevations. Using 238 interferograms over the area, the authors jointly estimated the deformations and the tropospheric delays. The results revealed that the estimated large-scale deformation and magma evolution from this study were much less than those from other studies, perhaps due to the fact that the atmospheric artifacts (ranging from -2.7 to +3.0 fringes) had been better accounted for in this study. Using ten SAR interferograms over Sakurajima volcano, Remy *et al.* [78] carefully investigated the relationship between the atmosphere-induced interferometric phases and the elevations, and found that the non-linear piecewise polynomial form of cubic splines was better in modeling the atmospheric delays than the linear models.

Chaabane *et al.* [81] suggested using the correlation between interferograms and that between atmosphere-induced interferometric phases and the elevation to correct for the atmospheric effects. In this approach, the global-scale atmospheric contribution is corrected by exploiting the correlation between the interferometric phases and the elevation, while the local atmospheric artifacts are corrected based on the correlation between interferograms containing a common acquisition. Test results with 81 differential interferograms covering the Gulf of Corinth (Greece) show that (1) the average uncertainty of the stacked deformation map has been decreased from ± 26 mm to ±12 mm, and (2) the RMS value of the differences between InSAR and GPS measurements at four stations has decreased from ±30 mm to ±19 mm after applying the correction.

The method of correlation analysis is advantageous in that no external data are needed. The method however strongly depends on the correlations between the deformations and between the atmospheric signals in different interferograms. Weak correlation may lead to insufficient atmospheric effect reduction.

### 5.3 Correction of Atmospheric Effects based on Pair-Wise Logic

The atmospheric signature in a SAR interferogram can be determined with the pair-wise logic method [21]. Atmospheric perturbations that are different from the pattern of local ground displacements can be identified by comparing interferograms spanning different time intervals. The method was used to find a 25×20 km kidney-shaped feature caused by ionospheric perturbations [8, 21]. Massonnet and Feigl [21] also found irregular patterns of up to three complete fringes resulted from tropospheric turbulences or increased water vapor over a 5×10 km area with this method. The qualitative nature of this method however makes it difficult to give exact values of the atmospheric effects. Hanssen [28] therefore suggested to sum or subtract two interferograms that use a common SAR image for removing atmospheric anomalies. The approach has also been referred to as the method of linear combination. It is effective when the atmospheric anomalies exist only in the common SAR image of the two interferograms.

### 5.4 Correction of Atmospheric Effects based on PSInSAR Technique

PSInSAR is a relatively new interferometic processing method [42, 79, 80]. It works on temporally stable coherent targets (permanent scatterers) only and can overcome the difficulties of coherence loss and atmospheric heterogeneities in conventional SAR interferometry. In PSInSAR, the atmospheric effects are modeled as linear phase ramps in the azimuth and the range directions for small ground areas or a more sophisticated model that includes, e.g., the linear ramps as well as the topography dependent term and the turbulence can be used for large rugged ground areas [82]. Parameters of an atmospheric model are estimated jointly with other unknowns such as the DEM errors and the LOS ground deformations at the permanent scatterers. The estimated atmospheric effects corresponding to each interferogram are then resampled onto the image grid with an interpolator and removed from the interferogram. Ferretti *et al.* [42, 79] reported that improved estimation of local topography and terrain motions was resulted over Ancona, Italy and Pomona, California with this method. Hooper et al. [80] modified the PSInSAR algorithms and applied the method to study the temporal and spatial deformation of volcanoes. A shortcoming of the method is that a significant number of SAR images over the same area, typically over 30, are needed to get reliable results.

### 5.5 Reduction of Atmospheric Effects with the Stacking Method

Stacking is a method that reduces the atmospheric effects on InSAR by averaging independent SAR interferograms. Assuming that atmospheric effects are uncorrelated between the interferograms, averaging *N* independent interferograms will reduce the atmospheric signals to $1/\sqrt{N}$ fold. The method was once regarded as the only viable solution to the problem of atmospheric effect mitigation [20]. Williams *et al.* [43] considered the method of interferogram stacking and that of atmospheric effect calibration with the assistance of external data (such as continuously operating GPS) to be complementary and suggested the two to be used simultaneously. Ferretti *et al.* [41] proposed a weighted averaging method by taking into account the spectral features of the thermal noise and the atmospheric component. Stacking in general degrades the temporal resolution of InSAR measurements, and the method works when there are only linear ground deformations as non-linear deformations can be lost in the process of stacking.

We have in this section looked through the various existing methods for mitigating the atmospheric effects on InSAR measurements. It should however be pointed out that in principle an optimal integration of some of the methods should yield the best results.

## 6. Conclusions

Atmospheric effects are one of the limiting error sources in repeat-pass InSAR measurements. They can introduce errors of over ten centimeters to ground deformations and of several hundred meters to DEMs measured with the conventional DInSAR method when considering the typical baseline geometries used. Studies have shown that atmospheric signals in SAR interferograms are anisotropic and non-Gaussian in distribution. The spectra of the atmospheric signals follow a power law distribution with the power exponent very close to -8/3. Various methods have been developed for mitigating the atmospheric effects on InSAR measurements based on external data such as ground meteorological observations, GPS data, satellite water vapor products such as those from MERIS and MODIS, and results from numerical meteorological modeling. These methods are typically able to reduce the atmospheric effects by about 20-40 percents. The other methods developed for mitigating the atmospheric effects are mainly based on simple data analysis or numerical solutions, including the pair-wise logic, the stacking, the correlation analysis, and the PSInSAR methods. Each of the methods developed has its pros and cons. The most suitable method should be chosen considering the number of SAR scenes acquired, the method used for InSAR processing, the atmospheric conditions (e.g., cloud conditions) and the external data available. Despite the progress already made in the research, further studies are still necessary in the area to develop more effective methods for the mitigation of the atmospheric effects.
